# Neonatal obstructive nephropathy induces necroptosis and necroinflammation

**DOI:** 10.1038/s41598-019-55079-w

**Published:** 2019-12-09

**Authors:** Bastian Popper, Marian Theodor Rammer, Mojca Gasparitsch, Teresa Singer, Ursula Keller, Yvonne Döring, Bärbel Lange-Sperandio

**Affiliations:** 1 Biomedical Center, Core Faciliy Animal Models, Ludwig-Maximilians university, 82152 Martinsried, Germany; 2 Institute of Pathology, School of Medicine, Technical University of Munich, 81675 Munich, Germany; 3 Dr. v. Hauner Children’s Hospital, Division of Pediatric Nephrology, Ludwig-Maximilians-University, 80337 Munich, Germany; 4 Institute for Cardiovascular Prevention, Ludwig-Maximilians-University, 80336 Munich, Germany; 5 Division of Angiology, Swiss Cardiovascular Center, Inselspital, Bern University Hospital, Bern, Switzerland

**Keywords:** Kidney, Kidney diseases, Apoptosis, Necroptosis

## Abstract

Urinary tract obstruction during kidney development causes tubular apoptosis, tubular necrosis, and interstitial inflammation. Necroptosis is a subtype of programmed necrosis mediated by the receptor-interacting serine/threonine-protein kinase-3 (RIPK3) and the pseudokinase mixed lineage kinase domain-like (MLKL). Necrosis induces inflammation and stimulates cell death in an autoamplification loop named necroinflammation. Here, we studied necroptosis and necroinflammation in obstructive nephropathy induced by unilateral ureteral obstruction (UUO) in neonatal C57Bl/6J mice. Ureteral obstruction induced tubular dilatation, tubular basement membrane thickening, cast formation, and increased expression of kidney injury molecule-1 (KIM-1). Morphological investigations showed either apoptotic or necrotic cells in the tubular compartment. Biochemical analysis revealed increased caspase-8 activity and upregulation of RIPK3 as well as phosphorylated-MLKL in UUO-kidneys. Pro-inflammatory cytokines (IL-1α, INF-γ, TNF-α) were upregulated following UUO. Taken together we show that necroptosis and necroinflammation are accompanied phenomena in neonatal kidneys with obstruction. These findings may help to develop novel strategies to treat congenital obstructive nephropathy.

## Introduction

Congenital obstructive nephropathy is a major cause of end-stage renal disease in infants and children^1,2^. Congenital obstruction of the urinary tract impairs renal development and reduces nephron numbers. Unilateral ureteral obstruction (UUO) in neonatal mice serves as a model for congenital obstructive nephropathy. UUO induces interstitial inflammation, apoptosis and necrosis, and leads to reduced nephron mass in the developing kidney with obstruction^3,4^.

For a long time apoptosis was considered being the only type of programmed cell death. Intensive research over the last decade revealed several other types of programmed cell death including necroptosis^5^. Necroptosis is present in different disease models, including ischemia reperfusion injury in the heart and kidney^6–8^. It is mediated by the receptor interacting protein kinase 3 (RIPK3) and the pseudokinase mixed-lineage kinase domain-like (MLKL)^9^. Induction of necroptosis occurs *via* binding of the tumor necrosis factor (TNF)-α to its receptor. TNF receptor activation usually induces apoptosis by initiation of the caspase 8 pathway^10^. By contrast, upon caspase 8 inactivation, necroptosis is favored. Thereby, RIP kinases and MLKL form a protein complex called the necrosome^11^. Initially both proteins RIPK1 and RIPK3 interact through RHIM (rip homotypic interaction motifs) domains. After activation of RIPK3 *via* RHIM-RHIM interactions, phosphorylated RIPK3 activates MLKL. Activated phospho-MLKL translocates to the cell membrane and forms a pore, which in turn leads to permeabilization and loss of membrane integrity^12^. Necroptosis and necrosis are two highly immunogenic forms of cell death that both induce inflammatory cell responses due to the synthesis of chemokines and/or the release of damage-associated molecular patterns (DAMPs)^13,14^. The auto-amplification loop of necrosis and inflammation, so-called necroinflammation, has been described in various kidney diseases^15^. So far, necroptosis and necroinflammation in the neonatal kidney with obstruction have not been studied.

In order to examine the contribution of necroptosis and necroinflammation in congenital obstructive nephropathy, we performed UUO in newborn C57Bl/6 J mice. We showed that UUO induces apoptosis, necrosis, and necroptosis in the developing kidney with obstruction. Key molecules of the necrosome (RIPK3 and MLKL) as well as inflammatory cytokines (IL-1α, INF-γ, and TNF-α) were significantly upregulated after obstruction. Ultrastructural analysis indicated that necrosis was primarily involved in proximal tubular cell death. In summary, our findings strongly suggest that necroptosis and necroinflammation contribute to the progression of renal tubular injury after UUO in newborn mice.

## Results

### UUO induces tubular injury

**Figure 1:**
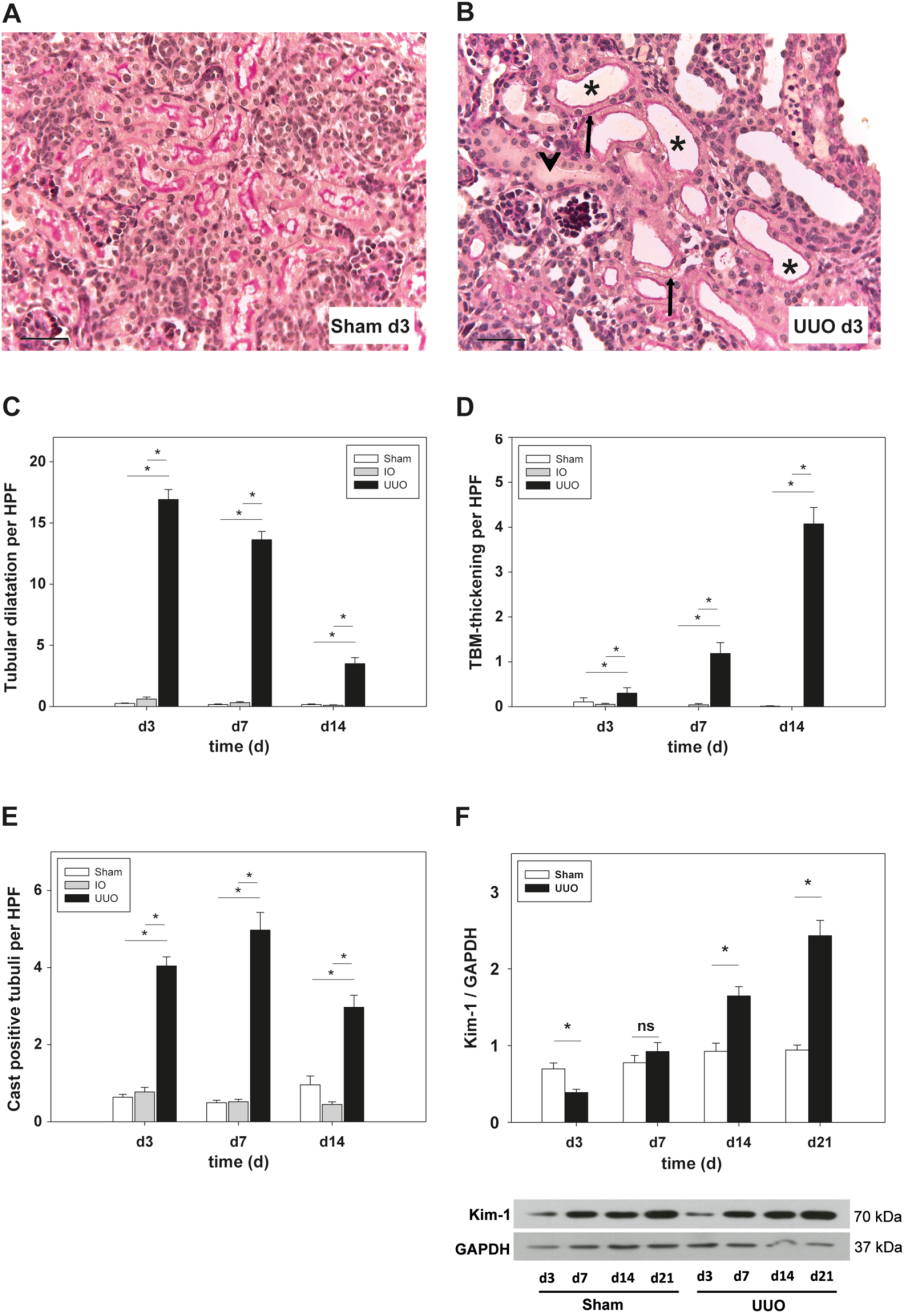
Histological investigation of PAS-stained kidney sections and Western blot analysis to detect renal injury following unilateral ureteral obstruction (UUO) in neonatal WT mice or sham-operated controls (sham) as well as intact opposite kidneys (IO). UUO surgery was performed on the second day of life (day 2). (**A**–**C**) Tubular dilatation increased within one day after UUO (asterisks) in comparison to sham-operated controls. Quantification revealed a significant increase at all time points investigated (p < 0.05). (**D**) UUO-induced thickening of the tubular basement membrane (arrows) which reached statistical significance at day 3 and peaked on day 14 in comparison to controls and IO kidneys. (**E**) Cast formation was quantified in UUO mice and controls. A significant increase in obstructed kidneys could be determined at all time points investigated. F. Whole kidneys were processed for Western blot analysis as described under Methods (n = 3/group). UUO induced protein expression of Kidney injury molecule (KIM-1) at day 14 and day 21 of life (p < 0.05). Bar = 100 µm. Magnification of 400x; *p < 0.05, ns = not significant, n = 8/group. Data are presented as mean + SEM.

To get first insight into how UUO impacts tubular morphology, we performed histological analysis of Periodic Acid Schiff (PAS) stained kidney sections of UUO mice at different time points (d3, d7, d14 of life). We compared our results with the intact opposite kidney (IO) of the same animal as well as with sham-operated (sham) control animals. Tubular dilatation peaked at day 3, which is 24 hours after ureter ligation. UUO-induced dilatation was most prominent in distal tubules and collecting ducts compared to proximal tubular segments of sham- and IO-kidneys **(**Fig. 1A,B**)**. Dilatation of tubular segments was 68-fold above controls in UUO-kidneys and remained significantly higher compared to controls and IO-kidneys for all time points investigated (p < 0.001). Moreover, we observed a decrease in tubular dilatation in UUO-kidneys at day 14 during disease progression (22-fold at day 14) **(**Fig. 1C**)**.

### UUO induces tubular basement membrane thickening

Tubular atrophy is generally hallmarked by thickening and folding of the tubular basement membrane (TBM)^3^. To study tubular atrophy in newborn mice, PAS-stained kidney sections were analyzed. UUO led to a significant increase of TBM thickening and TBM wrinkling in proximal and distal tubules at all time points investigated (p < 0.001) **(**Fig. 1D and Suppl. Fig. 1A**)**. Alterations of TBM integrity could be detected 24 hours after ligation and peaked on day 14 in UUO-kidneys compared to controls (sham-operated mice) and IO-kidneys.

### UUO induces cast formation

Injury of tubular epithelial cells can lead to detachment of tubular cells into the tubular lumen accompanied by tubular debris deposition and formation of protein aggregates including uromodulin^16^. So-called cast formation was investigated in PAS-stained kidney sections and transmission electron microscopy analysis (TEM) images from sham-operated and ureter-obstructed mice, as well as in IO-kidneys. UUO led to a significant increase of cast-positive tubuli at days 3, 7, and 14 compared to sham-operated and IO-kidneys (p < 0.05) **(**Fig. 1E and Suppl. Fig. 1B**)**. At day 7, we observed a 10-fold increase of cast positive tubuli in UUO-kidneys in comparison to controls. To complement our histological and electron microscopic analysis, we performed Western Blot analysis of Kidney injury molecule-1 (KIM-1), a marker for renal tubular injury^17^. KIM-1 expression increased significantly at day 14 and day 21 after UUO compared to sham-operated controls **(**Fig. 1F**)**.

### UUO induces tubular apoptosis

**Figure 2:**
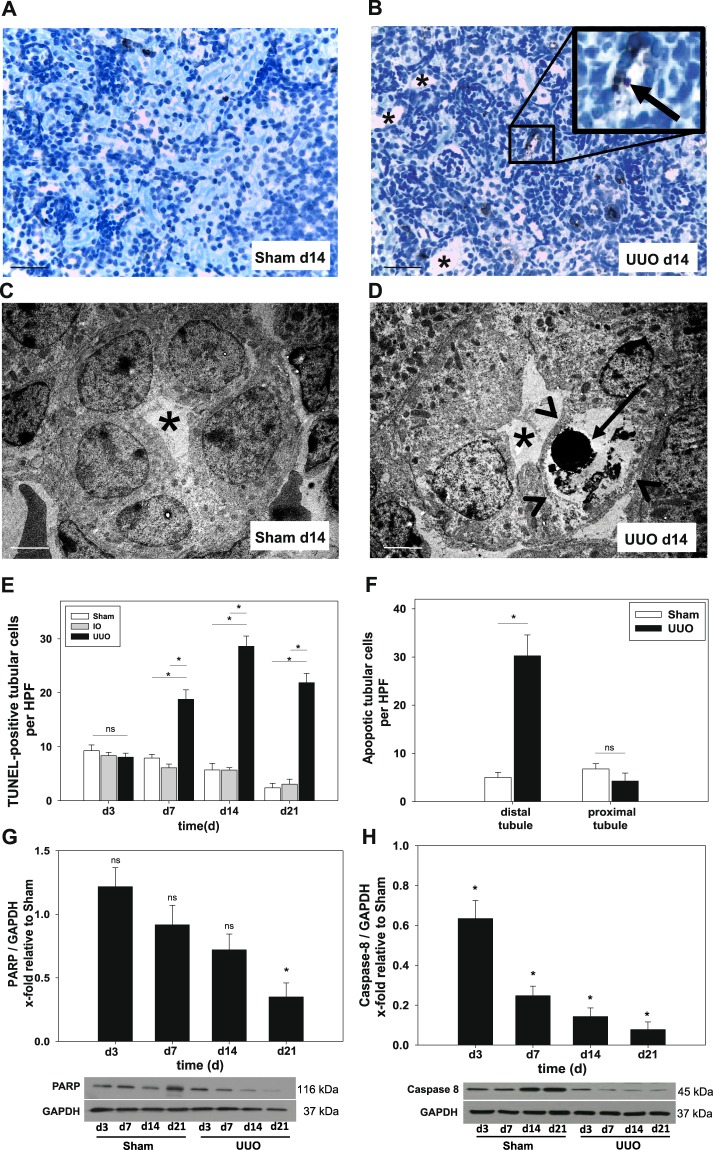
Morphometric, ultrastructural and western blot analysis to detect tubular apoptosis after UUO in neonatal WT mice. UUO was performed on the second day of life. (**A,B**) Apoptotic cells were detected by TUNEL staining in sections of sham-operated, IO- and UUO-kidneys at days 3, 7, 14 and 21 and were analyzed at x400 magnification. TUNEL-positive tubular epithelial cells (arrow) appeared in distal tubules. Representative pictures at day 14. Dilatated tubules are indicated by asterisks. (**C,D**) Transmission electron micrographs of sham-operated and UUO-kidneys showed chromatin condensation (arrow) whilst cell membranes appear intact (arrow heads) in distal tubules (asterisks). (**E**) Quantification revealed a significant increase in the number of TUNEL-positive cells in UUO kidneys compared to controls. (**F**) In depth analysis of the tubular compartment showed a preference of distal tubular epithelial cells to undergo apoptosis. (**G**) Whole kidneys were processed for Western blot analysis as described under Methods (n = 3/group). UUO induced cleavage of PARP and Caspase 8 (**H**) indexed as x-fold relative to sham-operated controls. A/B Bar 100 µm, C/D Bar 20 µm; *p < 0.05, ns = not significant, n = 8/group. Data are presented as mean + SEM.

Apoptosis is a major cause for renal tubular epithelial cell loss after neonatal UUO in mice^3^. Therefore, we tested for the internucleosomal DNA fragmentation, characteristic for apoptosis. By exploiting terminal deoxynucleotidyl transferase (TdT)-mediated dUTP-biotin nick-end labeling (TUNEL) of UUO-, IO- and sham-operated kidney sections at different time points (d3, d7, d14, d21), we found an significant increase in the number of TUNEL-positive tubular cells in the UUO-kidney from day 7 to day 14 (5-fold increase compared to day 3) (Fig. 2A–E). Of note, at day 21, the number of TUNEL-positive tubular cells decreased in UUO- as well as in control-kidneys (Fig. 2E). To confirm TUNEL analysis, we used transmission electron microscopy analysis to identify morphological hallmarks of apoptotic cell death in sham- and UUO-kidneys at day 14. Whereas sham-operated controls displayed normal tubular morphology (Fig. 2C), distal tubular segments of UUO-kidneys presented with rounded and pyknotic cells showing intact cell membrane and condensed chromatin (Fig. 2D). In addition, we found a significantly higher number of TUNEL-positive cells in distal tubuli but not in proximal tubuli (Fig. 2F). To further validate our findings, we performed Western Blot analysis of caspase 8 and Poly(ADP-ribose)polymerase (PARP), two well-known apoptotic markers^18^. Poly(ADP-ribose)polymerase (PARP) is an important DNA-binding enzyme involved in the initiation of apoptosis regulated by downstream targets of caspase 8. UUO-kidneys showed a steadily declining PARP expression in comparison to sham-operated controls, reaching statistical significance at day 21 (Fig. 2G). Caspase 8 is an important initiator caspase in the extrinsic pathway of apoptosis and regulator of RIPK3 activity^19^. Our results demonstrate a significant reduction of caspase 8 protein, whilst protein levels of cleaved caspase 8 increased in UUO-kidneys compared to sham operated controls during the disease progression (Fig. 2H and Suppl. Fig. 2A).

### UUO induces tubular necrosis and necroinflammation

**Figure 3:**
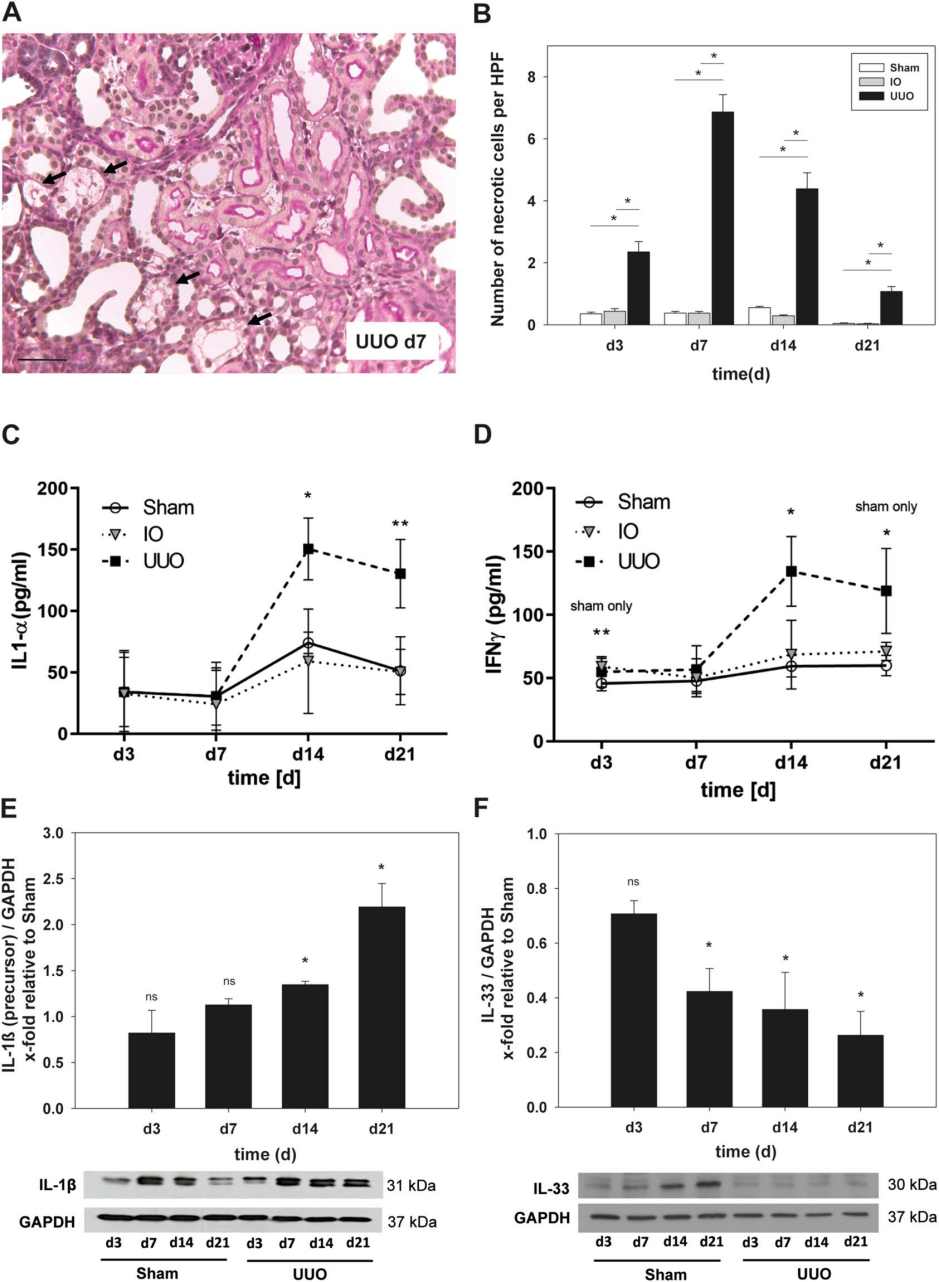
Morphometric, multiplex ELISA and western blot analysis were performed to detect tubular necrosis and necroinflammation after UUO in neonatal WT mice. Surgery (UUO) was performed on the second day of life. (**A**) PAS-staining of kidney sections of WT mice after UUO to detect tubular necrosis (representative picture at day 7 - arrows). (**B**) UUO induced significant increase of necrotic tubular cells with a maximum 5 days after ligation (d7 of life). (**C,D**) Supernatants of whole kidney lysates (n = 8/group) were analyzed by multiplex ELISA. Protein levels of pro-inflammatory cytokines IL-1α (**C**) and INF-γ (**D**) increased significantly in neonatal mice after UUO. (**E,F**) Whole kidneys were processed for Western blot analysis as described under Methods (n = 3/group). Results are indicated as x-fold relative to sham-operated controls. E. Expression of precursor form of IL-1β increased after UUO and peaked at day 21. F. Expression of IL-33 decreased after UUO. Bar 100 µm; *p < 0.05, ns = not significant. Data are presented as mean + SEM or +/− SD.

In the last decade several types of programmed cell death have been discovered^20^. Most of them share the same morphological alterations described for passive necrosis such as cellular swelling (oncosis), translucent cytoplasm, swelling of cell organelles, slight ultrastructural nuclear modifications (dilatation of the nuclear membrane and condensation of chromatin into small, irregular, circumscribed patches) and finally the disruption of the plasma membrane^11^. In PAS-stained sections of UUO-kidneys, tubular cells showed cytoplasmic swelling and loss of brush border integrity indicating that those cells underwent necrosis. Necrotic cells were mainly located in proximal tubular segments in UUO-kidneys compared to sham-operated controls **(**Fig. 3A). On day 3, we observed significantly more necrotic tubular cells in PAS-stained sections of UUO-kidneys in comparison to sham-operated controls or IO-kidneys **(**Fig. 3B and Suppl. Fig. 3A). The number of necrotic cells peaked at day 7 (18-fold increase in comparison to day 3) and declined on day 14 to day 21 **(**Fig. 3B**)**. The number of necrotic tubular cells in UUO-kidneys, however, remained significantly increased during the disease progression. To address whether the auto-amplification loop of necrosis and inflammation is part of the pathogenesis in UUO, we measured expression levels of proinflammatory cytokines that increase during necroinflammation^21^. Multiplex ELISA showed an upregulation of Interleukin (IL)-1α, Interferon (INF)-γ **(**Fig. 3C,D**)** and tumor necrosis factor–α (TNF-α) **(**Suppl. Fig. 3B**)** in UUO-kidneys at day 14 and day 21 **(**Fig. 3C,D**)**. Moreover, we complemented our ELISA findings with quantitative Western Blot analysis for Interleukin (IL)-1β. We found a significant increase in IL-1β precursor protein expression in UUO-kidneys compared to sham-operated controls **(**Fig. 3E**)** that peaked at day 21. Similar to IL-1β, Interleukin (IL)-33 is strongly associated with necroptosis^22^. However, in contrast to IL-1α and IL1-ß we observed a significant downregulation of IL-33 in neonatal mice with UUO **(**Fig. 3F**)**.

### UUO induces necroptosis in neonatal kidneys

**Figure 4:**
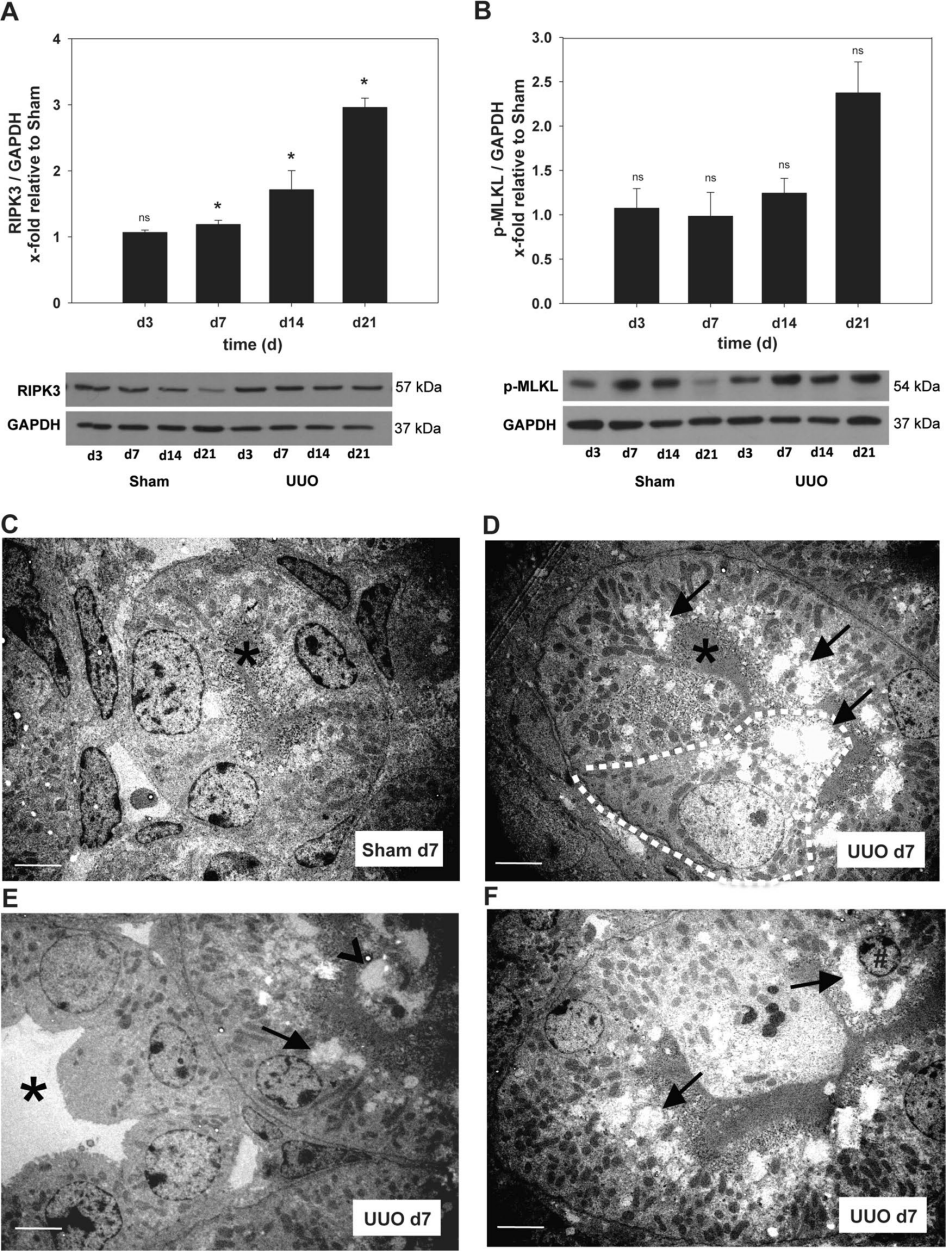
Western blot and ultrastructural analysis to detect necroptosis after UUO. Neonatal WT-mice were subjected to UUO or sham operation on the second day of life. (**A,B**) Whole kidneys were processed for Western blot analysis as described under Methods (n = 3/group). Expression of necrosome core proteins (RIPK3 and phospho(p)-MLKL) increased after ureteral ligation. UUO induced increased expression of RIPK3 (**A**) and p-MLKL (**B**) in comparison to controls. (**C**–**F**) Transmission electron microscopy analysis of sham- and UUO-kidneys. (**C**) Normal morphology of proximal tubular cells (asterisks indicates intact brush border). (**D**) Proximal tubular cells increase in size (dashed line) and protrude into the tubular lumen. Marked vacuolization of the apical cytoplasm is seen in almost all cells of the proximal tubule (arrow). (**E/F**) Distal tubular segments remain almost unaffected (asterisks) whilst neighboring proximal segments contain luminal cell detritus (arrow head) and vacuolized tubular cells with massive cell swelling (arrows) and chromatin condensation in the nucleus (# in F). Bars 20 µm; *p < 0.05, **p < 0.01; n = 8/group. Data are presented as mean + SEM.

Necroptosis is mediated by the RIPK3-MLKL signaling pathway that leads to plasma membrane disruption^9^. Therefore, we performed Western blot analysis to investigate RIPK3 and phospho-MLKL protein levels after obstruction **(**Fig. 4A,B**)**. Strikingly, we observed a significant and continuous upregulation of the necroptosis core protein RIPK3 in neonatal UUO-kidneys (3-fold increase above control at day 21) **(**Fig. 4A**)**. Next, we observed a concomitant upregulation of phospho-MLKL protein in UUO-kidneys compared to sham-operated controls. However, this effect did not reach statistical significance **(**Fig. 4B**)**. As a complementary approach, we used transmission electron microscopy analysis to investigate morphological changes of proximal tubular cells that are believed to be hallmarks of necrotic cell death^23^. Cells of the proximal tubular segment showed diffuse swollen electron lucent cytoplasm and vacuolization, whilst nuclei were mainly intact or shrunken or showed only mild chromatin condensation, indicating that these cells underwent necrosis **(**Fig. 4C–F**)**.

## Discussion

This study indicates a novel role for necroptosis and necroinflammation in the developing kidney with obstruction. Unilateral ureteral obstruction (UUO) in the neonatal mouse caused renal structural injury, induced key molecules of the necrosome (RIPK3 and phospho-MLKL) and generated inflammatory cytokines (IL-1α, INF-γ and TNF-α) in the neonatal kidney. Ultrastructural analysis indicated that necroptosis was primarily involved in proximal tubular cell death following UUO.

The concept of necroptosis is based on the interplay of various molecules that finally shape the necrosome^9^. The key mechanism is driven by RIPK3-mediated phosphorylation of the activation loop of MLKL, which in turn initiates plasma membrane rupture that ultimately leads to cell death^24^. Necroptosis itself is involved in cellular demise in several disease models^6^. In the kidney, tubular epithelial cells undergo necroptosis, especially after acute kidney injury^7,25^. Here we show that RIPK3 and phospo-MLKL were upregulated in a model of congenital obstructive nephropathy. Necroptosis reached an almost 3-fold increase two weeks after obstruction. Additionally, using TEM analysis we observed an increase in cell volume, organelle swelling, plasma membrane rupture and extensive intracellular vacuolization in proximal tubular segments of UUO-kidneys. In contrast, cells shrinkage, nuclear condensation and other characteristic features of apoptosis were mainly present in distal tubular segments. Those segment-specific findings are in line with previous reports, highlighting necrosis as the major form of cell death in proximal tubules^3^.

Morphological alterations in the tubular compartment play a fundamental role in the pathogenesis of congenital obstructive nephropathy. Dilatations of distal tubular segments and collecting ducts are early events following neonatal UUO^3^. Here we show a significant increase in tubular diameter one day after ureter ligation. TBM-thickening and wrinkling are reliable indicators of tubular injury and atrophy that mainly concern the proximal tubule^3^. Within one day after ligation, a thickened and wrinkled TBM was observed in UUO-kidneys, which is in line with previous results^3^. Furthermore, cell debris and dead tubular epithelial cells can detach into the tubular lumen and contribute to cast formation^16^. We observed cast formation in dilated tubules, suggesting tremendous cellular damage after UUO. Interestingly, tubular dilatation and cast formation tend to be early findings in neonatal UUO, whilst thickening of the TBM seems to be a response mechanism to prolonged shear stress and stretching of tubular segments later during disease progression. To also test for molecular markers of kidney injury, KIM-1 expression was measured in neonatal kidneys after UUO. KIM-1 is a promising biomarker for proximal tubular injury after ischemic stress^26^. Furthermore, KIM-1 can act as nonmyeloid phosphatidylserine receptor. In this function, it is able to transform renal epithelial cells into semiprofessional phagocytes. KIM-1 seems to be directly involved in phagocytosis of apoptotic and necrotic cells in injured tubules^27^. We showed a significant upregulation of KIM-1 in neonatal kidneys after UUO. Therefore, KIM-1 may be also a reliable marker for tubular damage in neonatal obstructive nephropathy. Using TUNEL staining we found a significant increase of cell death after UUO. A high majority of TUNEL-positive cells undergo apoptotic cell death. Nevertheless, the TUNEL-staining method itself has its limitations^28^. To confirm apoptosis as the major form of programmed cell death in the renal tubular compartment, we searched for typical morphological alterations of apoptosis in PAS-stained kidney sections. Apoptotic cells significantly increased and predominantly became visible in distal tubular segments starting at day 7. Further, we used transmission electron microscopy analysis to identify hallmarks of apoptotic cell death starting from day 7 until day 21 in UUO-kidneys. We identified significant more cells showing chromatin condensation, apoptotic bodies, and intact cell membranes in the distal tubular segments of UUO-kidneys. Those findings are in line with previous studies reporting that apoptosis was most frequent in the distal tubules and collecting ducts^29^.

On a molecular level, we showed an increase of apoptosis initiator caspase 8 activity on day 3 in UUO-kidneys compared to controls. That might indicate, that a high proportion of distal tubular cells undergo apoptosis early after ligation. Further, we observed reduced PARP protein level in UUO-kidneys compared to sham-operated mice. PARP cleavage is due to the activation of caspases of the apoptosis cascade^30^. Apoptosis is favored over necrosis in cells with high levels of ATP^31^. This might be the reason, why apoptosis is predominant in distal tubular cells after UUO. Proximal tubules are more prone to undergo necrosis due to oxidative damage^32,33^. UUO leads to significant ischemia and hypoxia in obstructed kidneys^34^. The ability to produce energy is limited in proximal tubular cells^3^. Consequently, UUO reduces proximal tubular cell mass, especially in neonatal mice^35^. We showed that necrosis was predominant in proximal tubules after UUO.

In contrast to rather immunologically silent apoptosis, regulated forms of necrosis are immunogenic^13^. The pro-inflammatory cytokines IL-1α and IL-33 are strongly associated with necroptosis^22,36^. Here we showed a significant upregulation of IL-1α, which might be a consequence of ongoing tubular necrosis in neonatal UUO-kidneys. IL-33 is involved in kidney remodeling and recruitment of immunogenic cells^37^. Surprisingly, IL-33 was downregulated in neonatal UUO-kidneys. By contrast, INF-γ, another immunomodulatory cytokine involved in tubular cell necroptosis and inflammation was upregulated following UUO^7^. Kopach *et al*. reported that INF-γ is able to downregulate pro-fibrotic IL-33 under certain conditions^38^. In the kidney, administration of INF-γ after UUO attenuated renal fibrosis in mice^39^. An interplay of IL-33 and INF-γ would explain the more pro-inflammatory and less pronounced pro-fibrotic state in neonatal UUO-kidneys, which also holds potential treatment options and needs to be studied in more detail. Noteworthy, despite its role in inflammation, INF-γ and TNF-α co-stimulate tubular cells to undergo apoptosis mediated by other members of the TNF-superfamily^40^. Interestingly, TNF-α and INF-γ increased simultaneously after UUO starting at day 14 after ligation. Both molecules might be involved in the initiation of the cell death machinery^41^. Whilst caspase 8 activation ultimately leads to apoptosis, TNF-α can trigger necrosis under certain conditions^42^. Those molecular findings are in line with our morphological investigations of UUO-kidneys showing a significant increase of apoptosis at day 14, whilst necrosis was observed earlier in the disease progression. We assume that necrosis in proximal tubular cells triggers cytokine release in response to UUO. Another cytokine, IL-1β, is expressed as a biologically inactive precursor molecule. The cleavage into the biologically active mature form depends on an initiation signal which can be mediated by caspase-1 and a complex called inflammasome^43^. Additionally the processing and release of mature IL-1β may depend on caspase-8-mediated apoptotic signaling^44^. UUO induced a two-fold upregulation of the IL-1β precursor molecule in neonatal kidneys compared to controls. Interestingly, an upregulation of IL-1β might be a consequence of increased RIPK3 activity, which is also MLKL independent^45^. Taken together we show that UUO induced expression of IL1-α and INF-γ in neonatal mice. These findings are in line with reports showing interstitial cell infiltration of macrophages in response to UUO^46,47^. Activated macrophages release cytokines like TNF-α, IL-1α and INF-γ, which in turn can induce cell death, tubulointerstitial fibrosis and inflammation^48,49^. Consequently, the interplay of inflammation and necrosis, called necroinflammation, seem to be accompanying phenomena also in the pathogenesis of neonatal obstructive nephropathy.

Necroinflammation and its inhibition may hold potential for therapeutic intervention in obstructive nephropathy. Several molecules are involved in necroptosis and other forms of regulated necrosis. The combined inhibition of several programmed cell death forms could be the best way to reduce damage in solid organ injury^50^. The effect of these potential inhibitors of cell death in UUO-kidneys has yet to be shown.

## Methods

### Experimental protocol

Two day old WT mice (C57BL/6J) with the same genetic background were subjected to complete left ureteral obstruction (n = 72) or sham operation (n = 72) under general anesthesia with isoflurane and oxygen inhalation at the second day of life as described before^3^ After recovery neonatal mice were returned to their mothers until sacrifice at days 3, 7, 14, and day 21 of life; n = 18 per group and time point. Representative photomicrograph after UUO-surgery is shown in Suppl. Fig. 4. All experiments were conducted according to national animal protection laws and the guidelines of animal experimentation established and approved by the Regierung von Oberbayern (Az 55.2-1-54-2532-118-11) and the Committee for Animal Experimentation of the University of Munich.

### Identification of tubular dilatation, TBM-thickening and Cast-formation

Kidney sections were stained with periodic acid Schiff (PAS) to investigate tubular dilatation in UUO and sham-operated mice as described previously^51^. Dilated tubules were identified by an enlarged tubular diameter in 20 sequentially selected fields at x400 magnification. Alterations of the tubular basement membrane (TBM) were determined as described previously^3^.

Deposits in the luminal compartment of all tubular segments were encountered as cast positive. The mean number of cast positive tubules was determined by counting the number of cast deposits in 20 sequentially selected microscopic fields of view at x400 magnification.

### Detection of cellular apoptosis

Apoptotic cells were detected by the terminal deoxynucleotidyl transferase (TdT)- mediated dUTP-biotin nick end labeling (TUNEL) assay as described previously^51^. Briefly, formalin-fixed tissue sections were de-paraffinized and rehydrated followed by incubation with proteinase K (20 µg/ml). After quenching, equilibration buffer was applied, followed by working strength enzyme (ApopTag Peroxidase *In Situ* Apoptosis Detection Kit, Millipore, Schwalbach, Germany). Cells were regarded as TUNEL positive if their nuclei were stained black and displayed typical apoptotic morphology. Apoptosis was calculated by counting the number of TUNEL positive cells in 20 sequentially selected microscopic fields of view at x400 magnification and expressed as the mean number of cells in 20 high power fields. The TUNEL assay does not only detect apoptotic cells but also necrotic cells^28^. Therefore, additional cellular parameters (e.g. nuclear morphology) and/or methods (e.g. TEM analysis, Western blot analysis of Caspase 8 cleavage) are needed to allow a precise characterization of cell death in UUO kidneys.

### Morphometric confirmation and segment localization of apoptosis

Hallmarks of apoptosis: chromatin condensation, cellular shrinkage, pyknotic nucleus or apoptotic bodies were used to quantify apoptosis in PAS-stained sections of kidneys of UUO mice and controls. The number of positive cells in distal tubules (no brush border) *vs*. proximal tubules (with brush border) was determined by analyzing 50 fields/kidney at x630 magnification (n = 8 per group).

### Ultrastructural analysis

For transmission electron microscopy analysis (TEM) the kidney cortex was cut into 1 mm³ samples and processed as described previously^25^. Samples were fixed with 2.5% glutaraldehyde in 0.1 M sodium cacodylate buffer, pH 7.4 (Electron Microscopy Sciences, USA) for 24 h at minimum. Thereafter glutaraldehyde was removed and samples were washed three times with 0.1 M sodium cacodylate buffer, pH 7.4. Postfixation and prestaining was done for 45 to 60 min with 1% osmium tetroxide (Electron Microscopy Sciences, USA). Samples were washed three times with ddH_2_O and dehydrated with an ascending ethanol series (15 min with 30%, 50%, 70%, and 90% respectively and two times 10 min with 100%). Subsequently, samples were embedded in Epon (Serva Electrophoresis GmbH, Germany). 60–70 nm thick ultrathin sections were cut at the Reichard-Jung Ultracut E microtome (Darmstadt, Germany). Ultrathin sections were collected on formvar coated copper grids (Plano, Germany) and automatically stained with UranyLess EM Stain (Electron Microscopy Sciences, USA) and 3% lead citrate (Leica, Wetzlar, Germany) using the contrasting system Leica EM AC20 (Leica, Wetzlar, Germany). Imaging was carried out using the JEOL -1200 EXII transmission electron microscope (JEOL, Akishima, Tokyo) at 80 kV. Images were taken using a digital camera (KeenViewII; Olympus, Germany) and processed with the iTEM software package (anlySISFive; Olympus, Germany).

### Multiplex ELISA for detection of necroinflammation cytokines

To determine concentrations of IL-1α, INF-γ and TNF-α in neonatal mouse kidneys, lysates of each mouse kidney (25 µl) were analyzed with the BenderMedSystems FlowCytomix Multiple Analyte Detection System as described previously^52^. In brief, this system uses fluorescent bead sets, each pre-coated with unique antibody specificity. Beads are first separated by size, then by quantities of a single fluorochrome. Monoclonal antibodies specific for one target (cytokines) are conjugated to the surface of the beads. When the protein of interest has bound, a secondary antibody and a detection fluorochrome are conjugated to enable cytokine detection. A serial dilution of the standards for the standard curve was added to the plate in duplicate. All samples were acquired and analyzed on a FACS Canto II (BD).

### Western immunoblotting

Kidneys of UUO and control mice were harvested on 3, 7 and 14 day of life (1, 5, and 12 days after obstruction) (n = 3 in each group) as described previously^4^. In brief, kidneys were homogenized in protein lysis buffer (Tris 50 mM, 2% SDS, 1 mM Na2VO2) containing proteinase inhibitor (Complete Mini, Roche Diagnostics GmbH, Penzberg, Germany) and benzonase (Novagen, Merck KGaA, Darmstadt, Germany) and centrifuged for 10 minutes at 16,000 x g. The protein content of the supernatants was measured using the BCA Protein Assay Kit (Pierce #23225). 15–20 micrograms of protein were separated on polyacrylamide gels at 160 V for 80 minutes and blotted onto PVDF-membranes (Millipore, Schwalbach, Germany) (80 mA/membrane, 90 min). After blocking antibody-specific for 2 hours in Tris-buffered saline with Tween-20 containing 5% nonfat dry milk and/or BSA, blots were incubated with primary antibodies 2 hours at room temperature or at 4 °C overnight. Rabbit anti Caspase-8 antibody (Cell Signaling Technology, #4927; 1:500), rabbit anti cleaved Caspase-8 (NB100-56116; 1:2000), rabbit anti RIP3/RIPK3 (both from Novus Biological, NBP1-77299; 1:2000), rabbit anti IL-1β (ab9722 1:1000), rabbit anti phospho-MLKL (both from Abcam, ab196436; 1:1000), rabbit anti KIM-1 (New East #21110, 1:1000), rabbit anti PARP antibody (Cell Signaling #9542, 1:1000), rabbit anti IL-33 (sc-98660; 1:1000, Santa Cruz, Heidelberg, Germany) were used for western blot analysis. GAPDH (DUNN Labortechnik H86540M) was used as an internal loading control and to normalize samples. Blots were washed with Tris-buffered saline with Tween-20 and incubated with horseradish peroxidase-conjugated secondary antibody for 1 h at room temperature. Immune complexes were detected using enhanced chemiluminescence method. Blots were exposed to x-ray films (Kodak, Stuttgart, Germany), the films were scanned and protein bands were quantified using the densitometry program Image J. Each band represents one single mouse kidney.

### Statistical analysis

Data are presented as mean values + SEM as not stated otherwise. Comparisons between groups were made using one-way ANOVA followed by the Student-Newman-Keuls or Tuckey post hoc test. Comparisons between two groups were made using the Students t-test for paired data. Statistical significance was defined as p < 0.05 as not stated otherwise. All statistics were performed with SigmaPlot (Systat Software GmbH, Erkrath Germany) or GraphPad Prism 6.0e (GraphPad Software, La Jolla California USA).

## Supplementary information

Supplementary Information

Supplementary Information

Supplementary Information

Supplementary Information

Supplementary Information
