# Supplementary material for: Neonatal obstructive nephropathy induces necroptosis and necroinflammation
Source: Sci Rep. 2019 Dec 9;9:18600. doi: 10.1038/s41598-019-55079-w (PMC6901532; doi:10.1038/s41598-019-55079-w)
Supplement: Supplementary file 1 — Supplementary Information [file 41598_2019_55079_MOESM1_ESM.pdf]

Neonatal obstructive nephropathy induces necroptosis and necroinflammation

Bastian Popper, Marian Theodor Rammer, Mojca Gasparitsch, Teresa Singer, Ursula Keller, Yvonne Döring, Bärbel Lange-Sperandio

Supplementary Figure 1 related to Figure 1

A

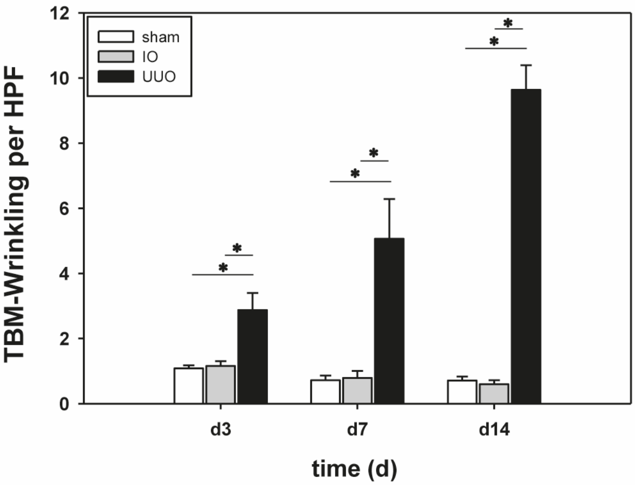

B

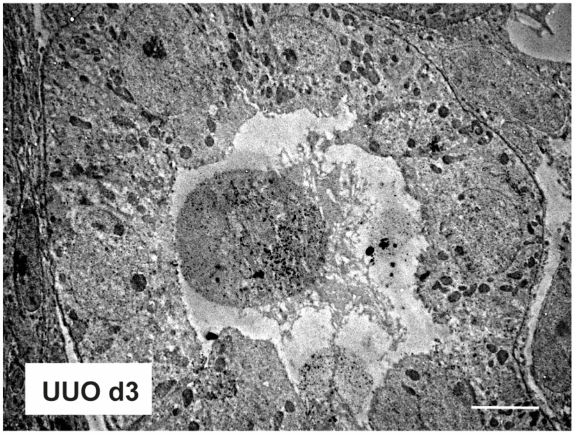

Cell dedritus in the lumen of a distal tubular segment (Cast formation)
