# Supplementary material for: Neonatal obstructive nephropathy induces necroptosis and necroinflammation
Source: Sci Rep. 2019 Dec 9;9:18600. doi: 10.1038/s41598-019-55079-w (PMC6901532; doi:10.1038/s41598-019-55079-w)
Supplement: Supplementary file 5 — Supplementary Information [file 41598_2019_55079_MOESM5_ESM.docx]

**Suppl. Figure 1**

A. UUO-induced TBM-Wrinkling that reached statistical significance at days 3, 7, and day 14 in comparison to sham-operated controls and IO kidneys. B. TEM image of Cast positive distal tubular segment confirming findings of PAS stained paraffin sections. Bar = 20 µm. * p < 0.05, n=8/group. Data are presented as mean + SEM.

**Suppl. Figure 2**

A. Whole kidneys were processed for Western blot analysis as described under Methods. Expression levels of cleaved caspase 8 (n=3/group) were evaluated in UUO-kidneys at days 3, 7, 14, and 21. Results are indicated as x-fold relative to sham-operated controls. * p < 0.05, ns = not significant. Data are presented as mean + SEM.

**Suppl. Figure 3**

A. Intact proximal tubular segments were evaluated by the integrity of the brush border in UUO-, IO- and sham-operated kidneys at days 3, 7 and 14. * p < 0.05, n=8/group. Data are presented as mean + SEM. B. Supernatants of whole kidney lysates were analyzed by multiplex ELISA. Protein levels of TNF-α (n=8/group) were evaluated in sham-operated, IO- and UUO-kidneys at days 3, 7, 14 and 21. * p < 0.05, ns = not significant. Data are presented as mean + SEM.

**Suppl. Figure 4**

Photomicrograph of the neonatal UUO-kidney at day 7 of life shows opened abdominal cavity and ligation of the left ureter. UUO was performed at the second day of life. Suture material is still in place. Left kidney is enlarged whilst the intact opposite kidneys appears normal.
